# Optimising Repeated Exposure: Determining Optimal Exposure Frequency for Introducing a Novel Vegetable among Children

**DOI:** 10.3390/foods10050913

**Published:** 2021-04-21

**Authors:** Klelia Karagiannaki, Christian Ritz, Louise Grønhøj Hørbye Jensen, Ellen Hyldgaard Tørsleff, Per Møller, Helene Hausner, Annemarie Olsen

**Affiliations:** 1 Section for Food Design and Consumer Behaviour, Department of Food Science, Faculty of Science, University of Copenhagen, Rolighedsvej 26, 1958 Frederiksberg C, Denmark; klelia.karagiannaki@outlook.com (K.K.); lghj@dtu.dk (L.G.H.J.); ellen_toersleff@hotmail.com (E.H.T.); p2moller@gmail.com (P.M.); helene.hausner@gmail.com (H.H.); 2 Department of Nutrition and Exercise Science, Faculty of Science, University of Copenhagen, Rolighedsvej 25, 1958 Frederiksberg C, Denmark; ritz@nexs.ku.dk

**Keywords:** repeated exposure, mere exposure, children, vegetables, taste, preferences, intervention

## Abstract

Fruit and vegetables are important components of a healthy diet, but unfortunately many children are not consuming enough to meet the recommendations. Therefore, it is crucial to develop strategies towards increasing the acceptance of this food group. This study aims to investigate the effect of different repeated exposure frequencies on fruit and vegetable acceptance using a novel vegetable, daikon, among 3–6-year-old children. One hundred and fifty-nine children participated in this study. Eight kindergarten teams were assigned to one of the following groups: Three different intervention groups with varying exposure frequencies, but all receiving seven exposures: Twice a week (*n* = 47), once a week (*n* = 32) and once every second week (*n* = 30), and a control group (*n* = 50). Liking and familiarity of daikon and other vegetables (cucumber, celery, celeriac, broccoli, cauliflower and beetroot) were assessed at baseline, post-intervention and two follow up sessions (3 and 6 months) to test for potential generalisation effects and observe the longevity of the obtained effects. Intake of daikon was measured at all exposures and test sessions. Results showed significant increases (*p* ≤ 0.05) in liking and intake of daikon for all three frequencies and the control group. Over the exposures, intake of daikon increased until the 4th exposure for all the groups, where a plateau was reached. No systematic generalisation effects were found. Repeated exposure was a successful approach to increase liking and intake of a novel vegetable with all exposure frequencies to be effective, and no particular exposure frequency can be recommended. Even the few exposures the control group received were found to be sufficient to improve intake and liking over 6 months (*p* ≤ 0.05), indicating that exposures to low quantities of an unfamiliar vegetable may be sufficient.

## 1. Introduction

The prevention of many chronic diseases has been shown to be reinforced by the consumption of a diet high in fruit and vegetables [1,2,3] as a result of their low energy but high fibre and important micronutrient content [1,4,5]. The recommended intake of fruit and vegetables for 4–10-year-old children in Denmark is 300–500 g per day, with half of the consumed quantity to consist of vegetables [6,7]. Pedersen et al. [5] showed that the consumption of vegetables of Danish children is lower than the recommended amount, a fact that is complemented by another survey of the same period which found that, even if the intake of vegetables had been improved, it was still under that of the guidelines [8]. An interesting finding was that vegetables were mostly consumed at lunch and dinner [9] compared to breakfast and in between meals, which were mostly high in sugar and fat [10]. The consumption of those high-calorie snacks instead of the rich in fibre and antioxidants fruits and vegetables, is one of the key risk factors of childhood obesity [11,12,13], thus it could be beneficial to swap those snacks with a vegetables in order to increase intake but also improve the nutritional value of the meal. Yngve et al. [14] found that the majority of vegetables eaten by Danish children are raw, while the study of Moore et al. showed that children who received vegetables three times per day consumed more servings on average, while the increased frequency was associated with larger variety [9]. Therefore, using a raw vegetable afternoon snack seems to be an efficient and easy choice.

The most crucial aspect of children’s fruit and vegetable intake seems to be their preferences [15,16] and as vegetables are not an innately preferred food, children must learn to like them in order to achieve healthier nutrition habits [17,18,19]. It is suggested that early age food habits [20,21,22], as well as their protective or unfavourable effects [23,24], are likely to be preserved in adulthood, hence it seems important to establish a preference for fruits and vegetables from a young age. There are several strategies that have been developed in order to increase children’s intake of fruit and vegetables, including flavour–nutrient learning, flavour–flavour learning and repeated exposure [25]. The latter supports that the preference for a specific food can be increased after repeated exposure to this initially unfamiliar food [25] and it has been successfully demonstrated [26,27,28,29,30]. Hausner et al. [31] showed that repeated exposure and flavour–flavour learning are useful strategies to change children’s attitudes towards a novel vegetable, proposing even a long-term result, with repeated exposure being the most effective method, which is in line with Remy et al. [32]. The lack of complexity seems to give the strategy of repeated exposure an additional advantage [33].

Humans are genetically predisposed to prefer some of the basic tastes (sweet and salty) and reject others (bitter and sour) [18,34]. This can explain why vegetables are not among the favourite foods for children, as they contain several bitter compounds. Moreover, a polymorphism in the TAS2R38 gene might provoke a variability in the perception of bitterness of the compound 6-n-propylthiouracil (PROP) among the population, and it has been demonstrated that children with a lower PROP threshold (super-tasters) were less likely to try cruciferous vegetables [35]. Consequently, children have to learn to like this food group [18,19], while it has been suggested that the optimum timing for this learning ability is from 4 months to 2 years old [36]. Parents are considered to be the basic factor forming their children’s eating preferences in the first year of life [18], while the heritability of neophobia is also an interesting topic under study [26]. For kindergarten children, in addition to taste, texture seems to also be important in forming their food preferences [19,37], with the desired texture attributes also relying on cultural elements [38].

*Neophobia*, defined as “fear of the new”, or the reluctance to try novel foods [39], also seems to be an obstacle for eating a varied diet [17,40]. Children with neophobia seem to consume a diet with less variety, that is, low mainly in vegetables, fruit and meat [41,42,43,44,45,46,47].

*Picky eating* is defined as the habitual rejection of either familiar or unfamiliar foods [48] which has, similarly to neophobia, the consumption of a diet low in variety as a consequence, usually poor in fruits and vegetables [49,50,51,52] as well as dietary fibre [50].

Both behaviours result in the toddler liking fewer kinds of fruits and vegetables but also trying fewer vegetables [53]. In general, food neophobia and picky eating lead to low diet diversity which is associated with decreased intake of micronutrients [54] and consequently the occurrence of nutritional deficiencies [55], and neophobic and picky children are reported to have a lower body weight [51,56]. However, it has been discussed that children with neophobic/picky behaviours might more easily accept some discretionary foods (energy dense and nutrient poor) due to their high palatability [57]. As they tend to reject healthy and non-energy-dense foods (such as vegetables) and often cover a larger part of their daily energy intake from these unhealthy, processed foods [58], it has been suggested that children with neophobic/picky behaviours can also be overweight and obese [57,59]. In other words, food neophobia and pickiness might be associated with both underweight and overweight or obese children [58], although there is a need for more evidence on this matter [57].

Food neophobia among toddlers has been linked to the pressure exerted from the parents on the child towards eating [60], while picky eating has been correlated with the dietary habits of the mother [59] and maternal feeding practices [61]. Actually, even though they are strongly correlated and share a common aetiology [62], picky eating is found to be mostly associated with environmental factors and experiences, compared to food neophobia which seems to also be influenced by individual parameters, such as genetic traits and temperament [45,62,63]. According to Nicklaus and Monnery-Patris, the traits of temperament are personal, rather stable, characteristics related to emotional and behavioural reactions [63]. The study of Moding and Stifter found that toddlers who are low on the *approach* dimension of temperament and tend to demonstrate negative effects and withdrawal responses to novel stimuli have increased chances to develop food neophobic behaviour during childhood [64]. Temperament includes inhibitory control, which is defined as the capacity to terminate, decrease or avoid a behaviour when instructed to do so, or when the circumstances are unfamiliar [65]. In the study of Rigal et al., the level of toddlers’ inhibitory control was identified as a determinant of the impact of repeated exposure, with the children that had high inhibitory control being able to overcome their dislike of unfamiliar foods more easily during a five-session exposure intervention [66].

Repeated exposure is an effective strategy for the reduction of neophobia [67,68] and it has been shown to also be negatively correlated with food pickiness [69]. It is defined as a replicated procedure where a stimulus is made approachable to an individual’s perception of it [70]. The repetition of presenting the stimulus to the individual is the key in order for the individual to change her/his attitude towards the stimulus [67,70]. As stated in the review of Mura Paroche et al. [71], repeated exposure is an important way for young children to learn about food and increase familiarisation to foods or textures. The strategy has been examined thoroughly in the context of changing children’s food preferences. An overview of exposure studies is provided in the Supplementary Materials (Tables S1–S6). From this, it is clear that studies vary substantially in setup, including exposure frequencies.

Most studies use a novel or a non-preferred food, aiming to increase the acceptance of it by repeatedly exposing children to it. Lakkakula et al. [27] proved that repeated taste exposures to poorly liked vegetables increased liking for most of them by elementary school children, while Maier et al. was able to demonstrate the same effect in infants [72]. According to Cooke [26], age is a determinant of the number of exposures needed to change the initial attitude, with older children requiring more exposures. This is in line with the study of Laureati, Bergamaschi and Pagliarini, which showed that a school-based exposure intervention was more effective in younger children [68]. As stated by Wadhera et al. [73], the effectiveness of this strategy on increasing liking for and intake of vegetables depends not only on age, but also on vegetable type. This is supported by Zeinstra et al. [29], who indicated that while repeated exposure is effective for increasing the intake of some vegetables, it might not be the best strategy for more familiar or blander tasting vegetables. Osborne and Forestell [74] studied if the type of exposure matters for the result, suggesting that repeated exposure, in the form of flavour exposures but also in the form of visual and informative cues of the stimuli through books, can be promising. In their study, children were exposed to two books, one about healthy eating and another one depicting drawings of fruits and vegetables, along with a taste exposure intervention. The exposure to books delivered positive results in increasing children’s consumption of fruits they had not tasted yet in the taste exposure. However, the authors did not observe the same for vegetables. Ahern et al. [75] showed that children’s liking of vegetables is related to the frequency of offerings of the vegetables, as they tend to like more the vegetables they are offered more often. This is in line with Grimm et al. [76] and Gregory et al. [77], who found that the less frequently the vegetables are offered during late infancy, the lower the intake during childhood. However, the impact of frequencies on the effectiveness of exposures is not yet clear as no more studies have investigated this subject to date, but a lot of exposure combinations seem to have succeeded.

On the other hand, it has been demonstrated that the liking and acceptance of a food might be reduced if it is consumed very often [26,27,28,29,31,33,34] and this can make the specific food less preferred. According to the definition of “*boredom*”, high familiarity with a food can decrease the desire to eat it [78]. This phenomenon is natural when using the strategy of repeated exposure as a consequence of the recurrent presentation of the stimulus [78]. According to Köster and Mojet [79], the level of complexity of the stimulus plays an important role in this process. The research editorial of Keller [80] discussed different intrinsic and extrinsic characteristics that influence the efficacy of the strategies used to improve children’s acceptance of vegetables and which can vary within the population. Among others, early flavour experience at the foetus stage or during breastfeeding, as well as the way the vegetable is prepared and presented during the intervention, are important parameters to consider when designing a more personalised and efficient approach.

A further aspect which may affect food preferences is *generalisation effects*, which are when acceptance of a novel food reinforces the transfer to other non-exposed foods with similarities to the first one. The presence of such effects is not well documented, but Ahern et al. [81] suggested that repeated exposure can generalise to other similar vegetables, also depending on the preparation technique.

Moreover, the association of foods with the environment contributes to the forming of food preferences [82] and children select food affected by social impact in a positive or a negative way [63,83]. An interesting point is that it is possible to reduce the level of neophobia with positive peer modelling, thereby altering food preference [84].

Culture is an important aspect that affects acceptance of novel food, as the determinants of rejection have been found to vary between populations. The cross-cultural study of Sandvik et al. found different texture drivers of liking a high-fibre biscuit among children of different nationalities [38], while Estay et al. demonstrated that children of different origin varied significantly in their liking scores for six vegetables [85].

### Aim of the Study

This study aimed at investigating different exposure frequencies and comparing their effectiveness on increasing the consumption of a novel vegetable by kindergarten children. The study also aimed at investigating whether there are generalisation effects from the exposures to other vegetables having more or less sensory characteristics in common with daikon.

The study was part of a larger study (Article “Optimising Repeated Exposure: Determining Optimal Stimulus Shape for Introducing a Novel Vegetable among Children”), sharing some experimental parts and groups, and in which a sensory profiling of the vegetables was made.

## 2. Materials and Methods

### 2.1. Study Design

Eight teams of kindergarten children were recruited for the study, six of each served as intervention teams and two served as control groups. Two of the intervention groups were exposed to the vegetable twice a week (2/week), the other two once a week (1/week) and the last two intervention teams were exposed on a bi-weekly basis. Assignment to the intervention groups was randomised. Three types of visits took place in the kindergartens: Two types of test visits and exposure visits. Pre- and post-control tests, conducted on two separate test days, were completed for all groups. The first visit included individual testing for liking, preferences and familiarity, while the second visit measured intake of the target vegetable. The six exposure groups underwent, in addition, seven exposure visits. The control intake session was set to be as close as possible to the exposure series, with the first exposure to start the week after the pre-test and the post-test to be completed the next week after the seventh exposure. The control groups did not receive any exposures. Follow-up sessions after 3 and 6 months, similar to the pre- and post-tests, were also completed for all eight teams. All the activities of the study were conducted in the subjects’ natural pre-school environment and in their mother language of Danish. The content of the different visit types is shown in Table 1.

### 2.2. Recruitment and Participants

Recruitment took place by inviting kindergartens in the greater area of Copenhagen to participate. Parents gave written, informed consent and children agreed to participate. After reviewing the protocol, the study was found not to require formal ethical approval by the Danish National Committee on Biomedical Research Ethics (ref. H-2-2011-FSP9). A reward of a DKK 50 (EUR ~ 7) gift certificate for a Danish toy store was given as an incentive to the increase response rate on questionnaires.

Four different kindergartens with eight groups and a total of 193 children aged 3–6 years participated. The characteristics of the children are given in Table 2.

### 2.3. Stimulus

Daikon (also termed Chinese radish) is a part of cabbage family (Brassicaceae) [86]. It shares many aroma compounds with cauliflower, which is also white, and broccoli, which are members of the same family [87]. Beetroot and celeriac come from two different families, not associated with Brassicaceae [88]. Celeriac has colour similarities with daikon, while beetroot has not. In that manner, the vegetables were selected to have different levels of similarities with daikon based on taste and colour.

Daikon was chosen to be the target vegetable, as it was expected to be unfamiliar and neither liked nor disliked. Previously, 9–11-year-olds have rated daikon as neutrally liked [89], while it was also found to be one of the least preferred vegetables [90], a fact that provides the possibility of an increase in intake. Grated daikon was predicted to be the most unfamiliar serving style; hence, this form was served during the exposure visits. On the other hand, round slices of daikon were served during the baseline and post-test visits, as well as for the 3-month and 6-month follow-up.

The generalisation effect was examined by rating the liking of the children for four other vegetables: Cauliflower, broccoli, celeriac and beetroot, due to their several shared characteristics with daikon. Cucumber and celery were used as “dummy vegetables” in order to practice the use of the 3-point hedonic smiley scale that was used in the study, considering cucumber as a generally liked vegetable and celery as an unfamiliar and rather disliked one [90].

The selection of the target vegetable and the procedure of the study were established after a small pilot study in which 16 children took part. Daikon was rated as moderately liked, with an average liking score equal to 2.1 (on a scale from 1 to 3, with 3 representing maximum liking of a stimulus), supporting the hypothesis that it would be a suitable vegetable to observe the improvements in liking and intake after the intervention.

### 2.4. Visits with Individual Testing

The individual testings were conducted in order to assess familiarity and liking of all the vegetables used in the study. These visits were carried out in the morning, before lunch time. The children were interviewed one-by-one by a trained assistant in a quiet area of the kindergarten. Familiarity was measured by showing each child a stick or floret of the assessed vegetable and noting on the questionnaire whether they had tasted the specific vegetable before. The 3-point hedonic smiley scale was used for the evaluation of liking and preference, as it has been previously demonstrated that it can be successfully used with 3-year-old children [91,92,93,94]. The scale was presented, explained and practiced with the children before the tasting. This was achieved by asking the children to first rank some favourite foods and, following that, using the dummy vegetables. Liking was measured by asking the child to taste the vegetable used for familiarity testing and score it on the smiley scale. The evaluation of the reference vegetables (broccoli, cauliflower and celeriac presented in random order) was first completed, before the assessment of beetroot and daikon, both served as random slices and following a randomised order. Every child had the choice not to try a vegetable or not to participate at all. All the answers of the children were noted down on a questionnaire.

### 2.5. Intake Visit

During this visit, the children were served 100 g of round sliced daikon in a pre-weighed plastic beaker as an afternoon snack, sitting all together. The subjects were instructed to eat as much as they liked and ask for a second serving of 100 g if they wished (maximum intake 200 g), while they were not allowed to share their beaker or comment on the food. In addition, it was possible to accompany this snack with water or milk. After the children finished eating the vegetable, the amount consumed was calculated by weighing the beakers on a scale with 0.1 g precision (Sartorius Gram TE15025, Sartorius AG, Göttingen, Germany).

### 2.6. Exposure Visit

The conduction of the exposure visit followed the very same procedure as the intake visit, with the beakers this time containing 100 g of grated daikon and without offering a second serving (maximum intake 100 g). The children were provided with a child-sized fork in order to eat the grated vegetable, and the headspace within the serving beakers was refreshed prior to serving to minimise smell. After each exposure visit, the beakers were weighed again on a calibrated precision scale to calculate the amount of the vegetable eaten by the children.

### 2.7. Samples

All vegetables were delivered by K.C. Frugt (Valby, Denmark) and stored at 5 °C. The different shapes were already processed (round sliced and grated) but they were slightly modified in order to achieve a perfectly round shape or to check for brown colouring. All vegetables were put in the beakers the same day of the kindergarten visit. The lid of each beaker was marked with the name of a child.

### 2.8. Questionnaire

At the beginning of the intervention, children’s parents were asked to fill out a 16-page-long questionnaire which included questions about the child and the parents. Fruit and vegetable consumption, general eating habits (“Children’s Eating Behaviour Questionnaire” [95]; “Comprehensive Feeding Practices Questionnaire” [96]), child’s liking and familiarity of the vegetables used in the study, as well as the Food Neophobia Scale adjusted for children [43,97], were covered.

### 2.9. Data Analysis

The data were analysed using the open-source software RStudio (Version 1.2.5019 © 2009–2019 RStudio, Inc., Boston, MA, USA), while Microsoft Excel (Version 16.26 (20091400) © 2020 Microsoft Corporation, Redmond, WA, USA) was used to structure the data, calculate the standard deviation (SD) and standard error of the mean (SEM) and prepare the plots. For the subjects of the exposure groups, an exclusion criterion was implemented; a child was excluded from the data analysis if he/she participated in fewer than four of the seven exposure sessions.

The influence on the liking of daikon and other vegetables of the different exposure frequencies was studied using the R packages *nlme* [98] and *multcomp* [99]. The first was used to run the linear mixed model (*lme function*) and the second in order to calculate the difference of the differences between and within the exposure and control groups for each session (*glht function)*. The initial model contained random and fixed factors, such as age and gender, as well as kindergarten institution, which were not found to be significant and thus they were excluded during the validation of the models. The reduced model used was simple and included only fixed factors and an interaction term for exposure frequency and timing.

The intake of daikon during the control sessions (pre-test, post-test, 3-month follow-up and 6-month follow-up) was associated with the various exposure frequencies using the same R packages mentioned above, *nlme* and *multcomp*. The linear mixed model was run using the function *lmer*, while the *glht* function was used to calculate the difference of the differences and extract the *p*-values. This model was used without reduction in the analysis, and it contained both fixed and random factors, as well as an interaction term for exposure frequency and timing. The fixed factors were baseline daikon liking, baseline familiarity to daikon, age and gender, and the random factors were participants’ ID number, group and kindergarten institution. The average intake of daikon during the seven exposure sessions was analysed similarly.

All *p*-values are taken from RStudio from the output of *glht function*.

## 3. Results

### 3.1. Subject Characteristics

As shown in Table 2, age and gender distributions were different in the four groups, but in the analyses, no significant age or gender effect was found. Thirty-four of the 193 children who took part in the study were excluded from data analysis as they were present in less than four exposures, leaving the data of 159 subjects for the analysis.

The information obtained from the questionnaires was in the end not used in the analyses, as the response rate of the parents led was too low (61%).

### 3.2. Test Visits

#### 3.2.1. Liking

**Figure 1:**
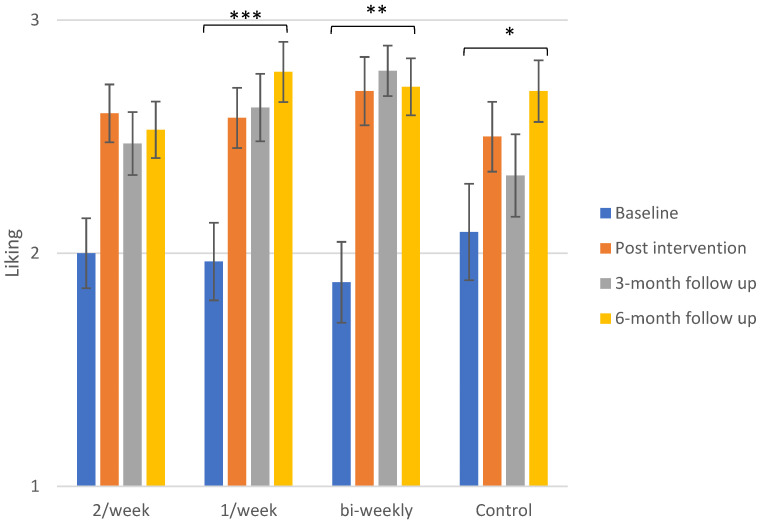
Mean liking scores ± SEM of daikon for the different exposure groups and control groups for the four control visits. The significance of the overall differences only (baseline to 6-month follow-up) for the groups of 1/week and bi-weekly exposure and the control group is depicted. The overall increase in liking of daikon was not found to be significant for the group that received daikon 2/week. The detailed differences and their statistical significance are given in the text. Significance levels: ‘***’: ≤0.001, ‘**’: ≤0.01, ‘*’: ≤0.05.

Overall, there is an increasing tendency for liking of daikon throughout the duration of this study, that is observed for all groups. Figure 1 illustrates the liking score for daikon between groups and within groups in the different control sessions (baseline, post-intervention, 3-month follow-up and 6-month follow-up). The significant differences that occurred from data analysis concern all the exposure groups, as well as the control group.

##### Control Group

Liking for daikon increased from baseline to post-intervention from 2.0 ± 0.2 to 2.5 ± 0.2, although this increase was not significant. A drop was observed at 3-month follow-up (2.3 ± 0.2) but the liking rose at 6-month follow-up, reaching its peak for the control group (2.7 ± 0.1), a value that is significantly higher than liking at baseline (*p* ≤ 0.05).

##### Exposure Groups

It seems that there are significant differences between daikon liking at baseline and most of the following control sessions for the children who received grated daikon in every exposure frequency, while the kinetics slightly vary among the groups.

For the subjects that received daikon twice per week, liking developed from 2.0 ± 0.1 of baseline to 2.6 ± 0.1 post-intervention (*p* = 0.001) and slightly dropped to reach 2.5 ± 0.1 at 3-month follow-up. The value of daikon liking for the 6-month follow-up remained rather stable (2.5 ± 0.1). The difference between baseline and 6-month follow-up was not found to be significant, as the *p*-value exceeded 0.05.

The exposure group that tasted daikon once per week expressed a steady increase in liking for the vegetable, as it rose from baseline (2.0 ± 0.2) to post-intervention (2.6 ± 0.1, *p* ≤ 0.001) and this remained almost stable at the 3-month follow-up (2.6 ± 0.2) but further increased at 6 months (2.8 ± 0.1). The increases recorded from baseline to 3- and 6-month follow-up were also found to be significant (*p* ≤ 0.001).

The subjects exposed to the vegetable every two weeks showed a larger increase in the liking for daikon, from 1.9 ± 0.2 at baseline to 2.7 ± 0.2 post-intervention (*p* = 0.005), that further rose to 2.8 ± 0.1 at 3 months and returned to 2.7 ± 0.1 at 6-month follow-up, with the overall difference (baseline to 6-month follow-up) found to be statistically significant (*p* ≤ 0.01).

In all groups, including the control group, the liking score was elevated at 6-month follow-up compared to baseline, indicating that even the low frequency and small quantity of the control group may be sufficient to significantly improve the acceptance of an unfamiliar vegetable. The changes between the initial control session and the 6-month follow-up were found to be statistically significant for all groups except for the group that received daikon twice per week. The liking of the vegetable in this group was effectively increased in the short term but was followed by a slight decrease, which was maintained in the long term (3 and 6 months), leading to a difference that was overall not significant. This pattern was somewhat also followed by the bi-weekly group concerning the 6-month follow-up, although the total increase was found to be significant for this group. A boredom effect could explain the first case, as the subjects were exposed to daikon more frequently, but not for the lower frequency of exposure. Overall, it seems that the highest liking value at 6-month follow-up was obtained by the subjects that were exposed once per week to the vegetable (2.8 ± 0.1), while it is important to note that the control group, receiving daikon just in the test sessions, achieved almost the same value at 6 months (2.7 ± 0.1).

#### 3.2.2. Intake

**Figure 2:**
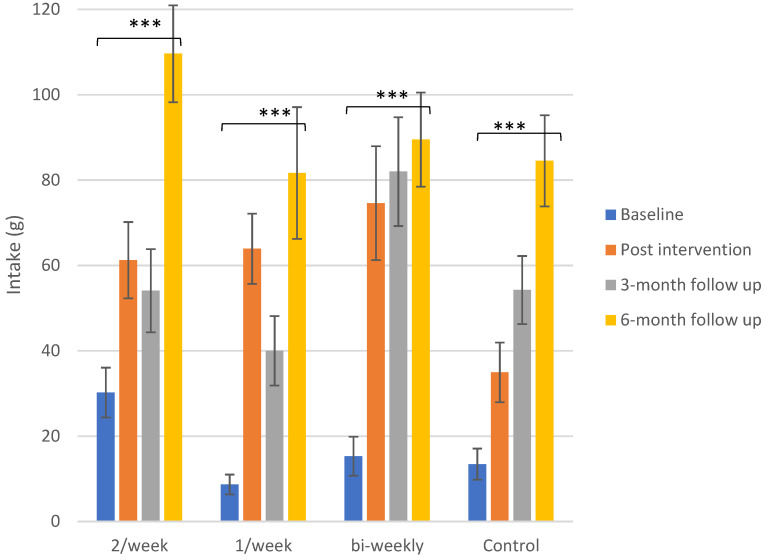
Mean intake scores ± SEM in g of daikon for the different exposure groups for the control visits. The significance of the overall differences only (baseline to 6-month follow-up) for the groups of 2/week, 1/week and bi-weekly exposure and the control group is depicted. The detailed differences and their statistical significance are given in the text. Significance levels: ‘***’: ≤0.001.

Intake in test sessions is shown in Figure 2. Intake of daikon was elevated from baseline to the following sessions in all groups, including the control group. Significant differences were found for the children who were exposed to the vegetable at any frequency but also for the control group, from baseline to post-intervention, baseline to 3-month follow-up and baseline to 6-month follow-up.

##### Control Group

The initial intake of 13 ± 4 g at baseline rose to 35 ± 7 g at post-test, 54 ± 8 g at 3 months and 85 ± 11 g at 6 months, with all these differences found to be significant (*p* = 0.002, *p* ≤ 0.001 and *p* ≤ 0.001, respectively).

##### Exposure Groups

For the exposure group that received daikon twice per week, intake increased from 30 ± 3 g at baseline to 61 ± 5 g post-intervention. The value slightly dropped at 3-month follow-up (54 ± 5 g) but reached a peak at 6-month follow-up (110 ± 6 g), which is the highest intake among all groups. The differences between baseline and all the other sessions were found to be statistically significant (*p* = 0.03, *p* = 0.02 and *p* ≤ 0.001, respectively).

The subjects that tasted the target vegetable once per week demonstrated the lowest initial mean intake (9 ± 2 g), which rose to 64 ± 8 g after the intervention (*p* ≤ 0.001), dropped at 3 months (40 ± 8 g) and peaked at 6 months (82 ± 15 g). Similarly to the other groups, the overall change was found to be statistically significant (*p* ≤ 0.001).

The children that were exposed to daikon once every two weeks showed a substantial increase in intake from baseline (15 ± 5 g) to post-intervention (75 ± 13 g) that continued to rise at 3 months (82 ± 13 g) and at 6 months (90 ± 11 g), with intake for all these sessions found to be significantly higher than baseline intake (*p* ≤ 0.001).

The total increase in intake from baseline to 6-month follow-up ranged around 70–80 g for all groups, indicating that the results of all frequencies and control were comparable in terms of quantity.

### 3.3. Exposure Visits

**Figure 3:**
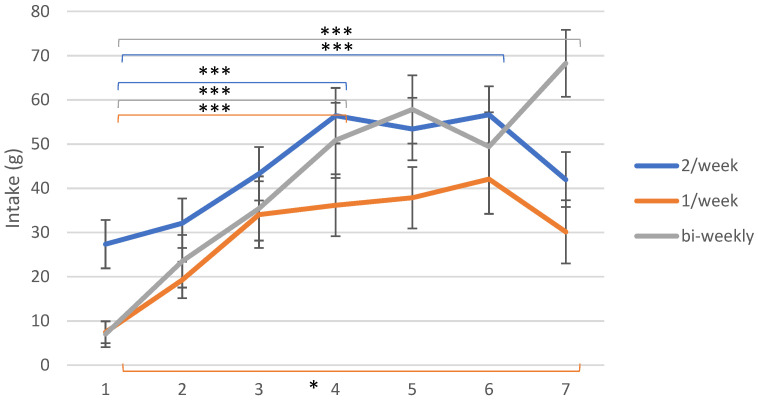
Mean intake scores ± SEM in g of daikon of the different intervention groups for each exposure session (1–7). The significance of the differences between selected sessions is depicted for all exposure groups. All groups showed a significantly higher intake at the 4th and the 7th exposure compared to the 1st one. The detailed differences and their statistical significance are given in the text. Significance levels: ‘***’: ≤0.001, ‘*’: ≤0.05.

All exposure groups demonstrated an increase in intake from the 1st to the 7th exposure, as shown in Figure 3, following quite similar tendencies.

For the exposure group that received daikon once per week, intake increased until the 6th exposure, when it reached a peak (mean intake of 42 ± 8 g of daikon). The analysis revealed significant differences between the 1st and the 3rd (*p* ≤ 0.001), 4th (*p* ≤ 0.001), 5th (*p* ≤ 0.001), 6th (*p* ≤ 0.001) and 7th (*p* = 0.02) exposure as well between the 2nd and the 3rd (*p* = 0.014), 4th (*p* = 0.006), 5th (*p* = 0.02) and 6th (*p* = 0.05) exposure.

Children exposed to daikon twice per week showed an increase in intake until the 4th exposure (56 ± 4 g) and afterwards a slight drop in the 5th exposure, and the average intake manifested a rather similar but highest level for the group at the 6th one (57 ± 8 g). The measurement that took place during the final exposure session was lower (42 ± 4 g), a value that was found to be significantly different to the ones of the 4th (*p* ≤ 0.001) and 6th exposure (*p* = 0.007), but not significantly higher than the initial exposure session. The data analysis for this group showed significant differences between the 1st and the 4th (*p* ≤ 0.001) and 6th exposure (*p* = 0.0015), as well as the 2nd and the 4th (*p* ≤ 0.001) and 6th exposure (*p* = 0.0015).

The group which received daikon bi-weekly demonstrated an almost steady increase until the 5th exposure and, after a decrease, a peak at the 7th exposure (68 ± 8 g), which is also the highest level of intake found among all groups and all exposure sessions. The differences between average intake of the 1st exposure were found to be significant compared to the 2nd (*p* = 0.01), the 3rd (*p* ≤ 0.001), the 4th (*p* ≤ 0.001), the 5th (*p* ≤ 0.001) and the 6th (*p* ≤ 0.001), as well as the comparison between the 2nd and the 3rd (*p* = 0.003), the 4th (*p* ≤ 0.001), the 5th (*p* ≤ 0.001), the 6th (*p* = 0.005) and the 7th (*p* ≤ 0.001) exposure. The difference between the 1st and the 7th exposure was also found to be significant, increasing from 7 ± 3 g to 68 ± 8 g of daikon (*p* ≤ 0.001).

For the two first groups (1/week and 2/week) we can highlight that intake in the final exposure was lower compared to previous exposures. On the contrary, children in the bi-weekly group consumed more daikon in the 7th exposure after demonstrating a significant reduction from the 5th to the 6th (*p* < 0.05) exposure session. This could indicate that more frequent exposure can lead to boredom, while a lower frequency of the vegetable actually led to the highest intake in the last exposure (68 ± 8 g compared to 30 ± 7 and 42 ± 4 g for 2/week and 1/week, respectively).

#### 3.3.1. Exposures 1–4

If we take into account the 4th exposure as a benchmark, we can observe that intake follows a continuous increase from the 1st until the 4th exposure for all the exposure groups, with the group that received daikon twice per week demonstrating the highest intake at that point.

#### 3.3.2. Exposures 4–7

After the 4th exposure, the average intake measurements follow a different pattern, with all the exposure groups reaching a plateau in different exposure sessions. The exposure groups of 1/week and 2/week recorded their peak level in the intake measurement at the 6th exposure and the group with a bi-weekly frequency at the 7th exposure.

To complement the above, we also need to evaluate intake during the follow up sessions, in order to conclude which frequency had an effect that lasted longer. At the 6-month follow-up, children exposed to daikon twice per week demonstrated the highest level of intake (110 ± 6 g), while liking at the same point showed the lowest score among the other groups (these differences in liking were not significant between groups). The children exposed to the vegetable once every two weeks had a mean consumption of 90 ± 11 g. The other two exposure groups showed a slightly lower intake for this session, with the intake of the control group at this session found to be slightly higher than that of the 1/week exposure group.

### 3.4. Generalisation Effects

Daikon is a member of the cabbage family (Brassicaceae), as are broccoli and cauliflower. Beetroot and celeriac were also used to study the possibility of a generalisation effect of the daikon exposures. They share some common aroma compounds, although they do not originate from the same family.

**Figure 4:**
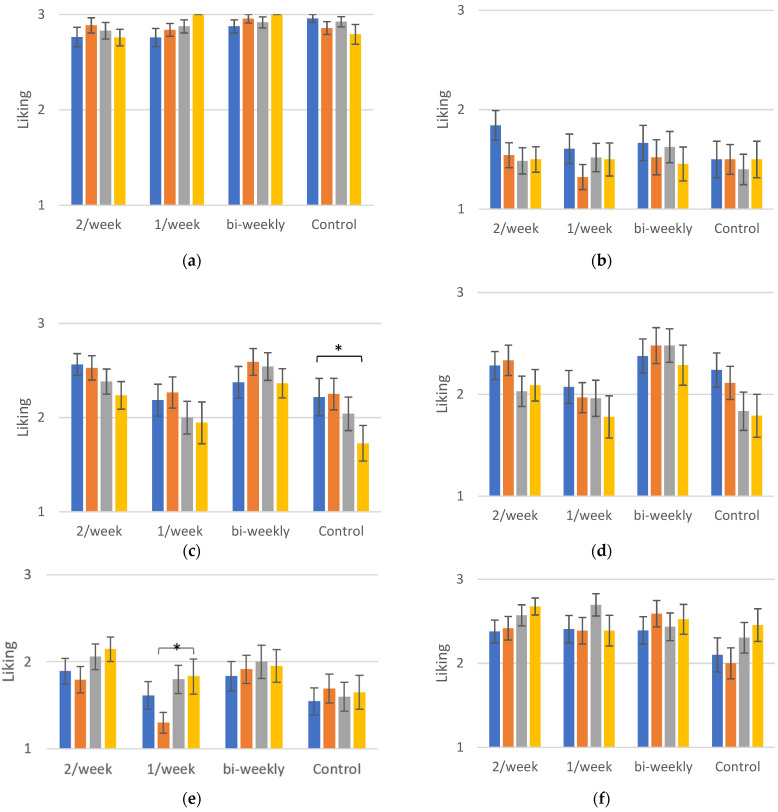
(**a**) Liking of cucumber for the different groups. (**b**) Liking of celery for the different groups. (**c**) Liking of broccoli for the different groups. (**d**) Liking of cauliflower for the different groups. (**e**) Liking of celeriac for the different groups. (**f**) Liking of beetroot for the different groups. Liking scores ± SEM of the different vegetables measured in control sessions for each group in order to investigate the presence of transfer effects. The significance of the differences between selected sessions is depicted for all exposure groups and vegetables. The detailed differences and their statistical significance are given in text. Significance levels: ‘*’: ≤0.05.

Cucumber and celery were used as dummy vegetables in order to test the three-point smiley hedonic scale. Cucumber is a generally liked vegetable, a fact shown by its liking score which almost reaches 3, while celery is a generally unfamiliar vegetable and its liking score remained below 2 in this study (Figure 4).

Figure 4a–f illustrate the liking of the different vegetables as measured in the control sessions. No significant differences were found for these vegetables in liking scores after the exposure of the children to Chinese radish, except for celeriac and beetroot. Celeriac was found to be liked significantly more at the 3-month follow-up (1.8 ± 0.2) compared to post-intervention (1.3 ± 0.1) for the group that was exposed to daikon once per week (*p* ≤ 0.05). There was also a significant difference in the liking of the celeriac at 3-month follow-up between the exposure group that received daikon twice per week and the control group (*p* ≤ 0.05). Concerning beetroot, liking was significantly higher post-intervention for the group that was exposed bi-weekly to daikon compared to the control group (*p* ≤ 0.05), while the latter showed a lower score at 3-month follow-up compared to the group that received daikon once per week (*p* ≤ 0.05). However, these results are not demonstrated systematically in this study and, therefore, we cannot conclude on a generalisation effect of exposure to daikon towards other vegetables.

The subjects of the control group also showed significant differences in liking of broccoli at baseline and 6-month follow-up (*p* ≤ 0.05) and post-test and 6-month follow-up (*p* ≤ 0.05). This is potentially connected to the small amounts of the vegetable that the children received during the control sessions, in line with the results for daikon that were discussed above.

## 4. Discussion

This intervention study aimed to explore the difference in the effectiveness of various exposure frequencies in improving liking and intake of a novel vegetable in kindergarten children.

### 4.1. Exposure Frequency

As shown in Table S1, there is a variety of intervention studies using the repeated exposure strategy towards increasing liking and acceptance of novel vegetables by children. The variability between these results of repeated exposure could be explained by different factors, such as the different serving styles and settings and the differences of initial liking of the vegetable, as well as the target food itself. The results of the present study are in line with many previous studies that found repeated exposure to be effective in toddlers and children [27,28,30,31,68,81,100,101,102,103,104,105,106,107,108,109]. The initial liking of the target vegetable was relatively low (with the mean score of most groups close to 2), which provided a potential window of improvement throughout the intervention period.

From the results of this study, it is seen that more frequent exposure led to more rapid changes, as the groups that received daikon once and twice per week demonstrated a steeper increase during the seven-exposure period which, nevertheless, reached a plateau and dropped by the 7th exposure. The groups with the more frequent exposure show a drop and peak levels of intake during the intervention slightly earlier than the group receiving daikon bi-weekly. This could suggest that more frequent exposures seem to deliver faster results, but indicating that the levels of boredom could also be more elevated in these cases. However, while the bi-weekly group demonstrates the highest level of intake in the final exposure session, the intakes reported at the 6-month follow-up for the different exposure groups are not significantly different (*p* > 0.05).

The kinetics of intake during the seven exposure sessions contradict the findings of Cooke, who suggested that in order to accept a novel vegetable, children need to be exposed to it 5–15 times [26], and the systematic review and meta-analysis of Nekitsing et al., who proposed at least 8–10 exposures [30]. In this study, one can observe that intake levels reach a plateau around the 4th–6th exposure for all groups, indicating that a smaller number of exposures might be a better idea in order to prevent boredom, a phenomenon that is well known to occur after a large number of exposures [34,110]. This is in agreement with the study of Caton et al., who found that as few as five exposures were adequate for toddlers to accept a novel vegetable purée [101].

It is interesting to combine the above with the pattern that the control group follows, as we can come to the conclusion that small quantities of the stimuli are sufficient to increase liking and intake. It should be noted that the control group of this study was not a “neat” control group, as it received some daikon on the control sessions (baseline, post-intervention, 3-month follow-up and 6-month follow-up) in order to evaluate liking and measure intake. The control group of course did receive a smaller amount of the vegetable in total as it was not part of the seven exposure sessions; thus, it seems that even this small amount and the low frequency of exposure to daikon had, as a result, an increase in both liking score and intake at 6 months. This shows that parents can see results in their child’s intake even if they present the vegetable in small quantities and at a flexible frequency.

Summing up, the boredom effect is not demonstrated clearly in this study in the long term, while it seems in general that all frequencies deliver comparable results.

### 4.2. Generalisation Effects

Some evidence for generalisation effects were seen. Grated daikon was used as the intervention stimulus, and sliced daikon during test days. Liking and intake increased for both stimulus types, which may be interpreted as generalisation effects taking place within the same stimulus type, i.e., the same stimulus may be served in different ways during exposures and still contribute to increased acceptance. However, no systematic generalisation effects were seen for the other types of vegetables. Similar findings were seen in the parallel study on the effect of the serving style of daikon Article “Optimising Repeated Exposure: Determining Optimal Stimulus Shape for Introducing a Novel Vegetable among Children”). Previous studies investigating generalisation effects did not show consistent findings, thus the effectiveness of repeated exposure in increasing liking of other similar foods is not yet clear. Ahern et al. [81] observed an increase in children’s acceptance and intake of a different stimulus that shared similar characteristics to the ones they had been exposed to, which is in agreement with Birch and Loewen [111,112]. On the other hand, Olsen et al. and Hetherington et al. [89,113] could not demonstrate the effect. The vegetables used in the study were chosen strategically to have shared characteristics with the target vegetable, in agreement with Birch et al. [82], who suggested that the generalisation effect is more evident in similar stimuli. Nevertheless, the results of the present study were unable to show a concrete generalisation for the control vegetables.

### 4.3. Limitations

A limitation in this study is potential increases in intake due to children growing throughout the duration of this study and thereby increasing their consumed amounts. Although this applies to all experimental groups, it is particularly relevant for the group with exposures every second week, as their exposure period covers a longer time period. Including an additional age-matched control group to be tested only before the intervention and at 6-month follow-up could be a potential solution to this limitation for future studies.

As demonstrated in the sensory profiling study Article “Optimising Repeated Exposure: Determining Optimal Stimulus Shape for Introducing a Novel Vegetable among Children”), the different shapes of daikon show distinct sensory characteristics. The round shape used in the intake evaluation was found to be sweeter than the grated daikon used in the individual testings and the exposure sessions and which was characterised as bitter and pungent. This variability of the sensory attributes could have affected the consistency of the results between the different types of visits. Furthermore, the use of round daikon at test visits may have reduced recognition somewhat and resulted in lower scores for liking and intake than might have been seen if the test stimulus was grated daikon, as used during exposures.

The control group tasted both grated (individual testings) and round (intake evaluation session) daikon during the control visits, presenting a low frequency of exposure to the vegetable. This fact led to the positive conclusion that even small amounts and a low frequency and tasting of an unfamiliar vegetable can deliver results in terms of children’s acceptance.

The maximum intake level during the control visits (200 g) was different compared to the exposure visits (100 g), a fact that complicates the comparison of the amounts of daikon consumed. The average intake at control visits might have been increased due to those children who ate more than one portion of daikon.

Finally, it should be noted that other findings may be obtained using other exposure frequencies, stimulus types and age groups.

## 5. Conclusions

The findings in this study on optimum exposure frequency to an unfamiliar vegetable were not clear. All exposure frequencies lead to an increase in liking and intake of daikon, with the more frequent ones possibly displaying more rapid outcomes but also reaching a plateau in intake faster than a low exposure frequency. Due to the positive outcomes of the control group, even low frequencies may provide positive changes in acceptance and the exposed quantity does not necessarily need to be large. No evidence for systematic generalisation effects was found.

## 6. Implications

The findings from this study can easily be applied and implemented at home and in institutional settings, as it seems that repeated exposure is indeed an effective strategy to increase liking and intake of a novel vegetable for children 3–6 years old. Among the frequencies tested, they were all sufficient, and none in particular can be recommended as optimal. This supports a large amount of flexibility among parents and caregivers to introduce unfamiliar vegetables at a frequency convenient to the cooking schedule, time and place, without adhering to particular frequencies to do so. Interestingly, even a few infrequent exposures given over a long time seem to be sufficient; a promising result for parents who want to succeed in vegetable acceptance of their children with a few exposures. It is important to keep in mind that the home food environment may act as a mediator of such intervention effects, with caregivers’ fruit and vegetable intake playing an important role as well [114].

Since generalisation effects were not found, parents and caregivers wanting to increase liking and acceptance of novel vegetables should bear in mind that this requires presenting each vegetable on its own, as it cannot be expected that exposing children to one vegetable will generalise to others.

## Figures and Tables

**Table 1:** Overview of the content of the different visit types.

Type of Visit	Groups	Test	Vegetable
Pre-test/baseline measurement	All	Individual testing	Familiarity and liking: Broccoli, Cauliflower, Celeriac, Daikon (round), Beetroot (round)
	All	Intake (200 g)	Daikon, round
Exposure twice a week	2/week	Seven exposures (100 g)	Daikon, grated
Exposure once a week	1/week	Seven exposures (100 g)	Daikon, grated
Exposure once every second week	Bi-weekly	Seven exposures (100 g)	Daikon, grated
Post-test	All	Intake (200 g)	Daikon, round
	All	Individual testing	Familiarity and liking: Broccoli, Cauliflower, Celeriac, Daikon (round), Beetroot (round)
3-month follow-up	All	Intake (200 g)	Daikon, round
	All	Individual testing	Familiarity and liking: Broccoli, Cauliflower, Celeriac, Daikon (round), Beetroot (round)
6-month follow-up	All	Intake (200 g)	Daikon, round
	All	Individual testing	Familiarity and liking: Broccoli, Cauliflower, Celeriac, Daikon (round), Beetroot (round)

**Table 2:** Subjects’ characteristics.

	Control	2/Week	1/Week	Bi-Weekly
*n*	50	47	32	30
Girls/boys	23/27	30/17	14/18	15/15
Age ^1^ ± SEM	51.8 ± 2.0	55.0 ± 0.9	53.8 ± 2.1	53.8 ± 1.4

^1^ Age is presented in months.

## Data Availability

Data is not avaible for sharing.

## Supplementary Materials

The following are available online at https://www.mdpi.com/article/10.3390/foods10050913/s1, Table S1: Studies using mere exposure to increase intake/liking in infants (<12 months). The studies are sorted according in ascending to publication year. Success-column: an evaluation of the effect of mere exposure; +: clear effect, (+): some effect but not clear, -: no effect. Table S2: Studies using mere exposure to increase intake/liking in toddlers and children (>12 months -17 years). The studies are sorted according in ascending to publication year. Table S3: Studies using mere exposure to increase intake/liking in young adults and adults (>18 years). The studies are sorted according in ascending to publication date. Suc-cess-column: an evaluation of the effect of mere exposure; +: clear effect, (+): some effect but not clear, -: no effect. Table S4: Studies using mere exposure to increase intake/liking (recalled/retrospective data). The studies are sorted according in ascending to publication date. Success-column: an evaluation of the effect of mere exposure; +: clear effect, (+): some effect but not clear, -: no effect. Table S5: Studies using visual mere exposure to increase intake/liking). The studies are sorted according in ascending to publication date. Success-column: an evaluation of the ef-fect of mere exposure; +: clear effect, (+): some effect but not clear, -: no effect. Table S6: Studies using hands-on mere exposure to increase intake/liking. The studies are sorted according in ascending to publication date. Success-column: an evaluation of the effect of mere exposure; +: clear effect, (+): some effect but not clear, -: no effect.
