# Supplementary material for: Optimising Repeated Exposure: Determining Optimal Exposure Frequency for Introducing a Novel Vegetable among Children
Source: Foods. 2021 Apr 21;10(5):913. doi: 10.3390/foods10050913 (PMC8143368; doi:10.3390/foods10050913)
Supplement: Supplementary file 1 [file foods-10-00913-s001.zip › foods-1153586-supplementary.pdf]

Table S1: Studies using mere exposure to increase intake/liking in infants (<12 months). The studies are sorted according in ascending to publication year. Success-column: an evaluation of the effect of mere exposure; +: clear effect, (+): some effect but not clear, -: no effect.

**INFANTS: PRE- AND POSTNATAL (< 12 MONTHS).**							
**Exposure Frequency**	**Number of Exposures**	**Exposure Stimuli**	**Serving Style**	**Age of Participants**	**Results**	**Success**	**Author(s) and Year**
One full day – 24h	24 h	Artificial commercially available odorant: Cherry essential oil and white ginger perfume essence.	15 drops of exposure odour were placed on a clean 10- x 10 cm gauze pad. The odorized pad was placed behind and slightly above the infant's head.	New-born – 5-21 ½ h after birth.	Female infants displayed preferential orientation to the exposure odour. Males did not.	(+)	(Balogh and Porter, 1986)
1/day	10	Factory puréed peas (with or without salt), puréed green beans (with or without salt).	Jar of 71 g with no indication of salt/no salt on label.	4-6 months	All groups increased their intake of the target stimuli significantly and no increase was seen in the control food. Increased intake for the other version of the same vegetable from pre- to post-test was also seen.	+	(Sullivan and Birch, 1994)
1/day	10	Commercially available puréed peas and puréed banana ("target" food)	Babies were fed by their parent. Two containers (113 g) were transferred to standard serving containers provided by the experimenter. "Same" food: the other manufacturer's preparation of the target food. "Similar" food: from the target food category (other fruits or vegetables for bananas and peas respectively). "Different" food: the target food of the other group.	4-7 months	Significant increase of "same" and "similar" and "target" food. Increase of "different food" was unchanged. No generalisation effect was seen.	+	(Birch *et al.*, 1998)
1/day	12	Commercially available puréed carrot, puréed po-	The purées were presented in jars.	4-5 months	Intake of the exposure stimulus increased significantly for all groups. Intake of carrots increased for the group	+	(Gerrish and Mennella, 2001)

	(day 1, 11 and 12 laboratory, day 2-10 home exposure)	tato or purée of three different vegetables (peas, potatoes, squash)<br>Puréed chicken and pureed carrot acceptance were evaluated after the exposures.	Mothers were sent a bib and spoon to use when feeding. Babies were fed by their mother wearing a mask.		exposed to carrot and the group exposed to different vegetables. Generalisation: Intake of chicken increased significantly only for the group exposed to different vegetables.<br>Further the study showed that previous daily experience with fruit enhanced initially acceptance of carrots.		
4/week	12 x 2	Carrot juice and water (during the last trimester of pregnancy and then again during the first 2 months of lactation)	Mothers: Bottles of mineral water, 300ml, and cartons of frozen organic carrot juice, 300ml, and instructions regarding the time of day (10 am to 2 pm) to drink the beverage.<br>Cereal prepared with water (1-part cereal, 1-part water) and cereal prepared with carrot juice (1-part cereal, 1-part carrot juice) for testing preference.	Infants, Approximately 6 months	Infants who mothers drank carrot juice either while pregnant or breastfeeding showed fewer negative facial expressions to carrot-flavoured cereals than infants whose mothers only drank water.	+	(Mennella, Jagnow and Beauchamp, 2001)
Daily	1 ½- 2 ½ months	Milk formula vs. protein hydrolysate formula.<br>Different solutions of apple juice as test food.	10 ml juice served in opaque tumbler cups in a laboratory setting.<br>6 sour apple juices (added citric acid) and 6 sweet apple juices (added sugar or water for dilution).	Infants (4-7years)	Younger children who were fed protein hydrolysate formulas preferred higher levels of citric acid in juice when compared to older children who were fed similar formulas.<br>No difference was observed between the groups for sweet preference.	(+)	(Liem and Mennella, 2002)
1/day	8	Commercially available green bean purée	Mothers were given jars of purée d infant food for the home-exposure period. Each jar was labelled with the date on which the contents of that particular jar should be fed.<br>Infants were the contents of 1 jar of puréed green beans until	4-8 months	Exposure did increase intake significantly	+	(Forestell and Mennella, 2007)

			the infant either refused the food on ≥ 3 consecutive occasions or finished the content of the jar.				
Every second day	8	Individually disliked vegetable purée (factory produced purees in jars)	Infant fed with spoon. Three 130g pots of the purée were available for each meal.	7-10 months Follow up at 15 months, 3 and 6 years	Exposure did increase intake. 71% of the infants ate the same amount of a liked and the disliked purée after 7 exposures. Intake of the initially disliked puree rapidly increased over days, and by the final exposure was equal to that of vegetable purees that were initially liked. These effects of repeated exposure were still present nine months later. By the sixth exposure day, liking scores were similar. Follow up: At 15 months, the initially disliked vegetable that became accepted after repeated exposure was still liked and eaten by 79% of the children. At 3 years, the initially disliked vegetable was still liked and eaten by 73% of the children. At 6 years, observations in an experimental setting showed that children who had been breast-fed and children who had experienced high vegetable variety at the start of weaning ate more of new vegetables and liked them more	+	(Maier *et al.*, 2007) (Maier-Nöth *et al.*, 2016)
1/day	8	Fruits: pears or another target fruit Vegetables: green beans or a variety of vegetables	Stage 1 puréed pears Stage 2 puréed green beans Mothers fed their infants using the same type of bib and spoon used prior to the study. If the	4-9 months	Infants repeatedly exposed to either pears or a variety of fruits consumed significantly more pears after the 8-day home exposure.	+	(Mennella *et al.*, 2008)

			baby finished the contents of the jar, the jar was replaced with another such that the maximum amount of food offered was 220 g.		Infants consumed 3.2 ± 0.6 times more green beans, 3.6 ± 1.7 times more carrots, and 5.1 ± 2.2 times more spinach after the 8-day home exposure. Repeated dietary exposure to pears and green beans resulted in babies tending to eat more of that particular food		
Every second day	10	Caraway flavoured potato purée	Portions of 125 g purée were provided for each exposure meal. Infants were fed with a spoon and mothers were instructed to feed their infants at the same time on all exposure days.	5-8 months	Increased intake after the exposure period	+	(Hausner *et al.*, 2009)
1/day	4 different stimuli x 6 consecutive days	Four novel vegetable purée s: green beans, peas, squash, and carrots	The food was commercially available and were delivered to their homes with labels indicating the day on which they were to be presented to the infant. Patents were asked to feed each week's food at a similar time each day.	8-10 months	They found a 10–30% increase in intake of all vegetables (except carrots) from pre- to post-intervention.	+	(Paul *et al.*, 2011)
2/week or 3/week	10	Artichoke purée (target) Carrot purée (control)	Two 100-g jars of heated artichoke purée were presented for either lunch or dinner (same time every exposure). During each experimental meal the parents should not give another vegetable purée and also not give other artichoke- based foods between pre- and post-exposure.	4-8 months	Between pre- and post- exposure, intake and liking of the basic artichoke purée significantly increased. Repeated exposure was effective at increasing intake of basic artichoke purée in the short term. After the 3-month follow up the artichoke intake decreased but the learning effect can be still observed.	+	(Remy *et al.*, 2013)

1/day	19	Vegetables: green bean purée or artichoke purée<br>Fruits: apple purée or plum purée	At the beginning of the study on days 1 and 2 and at the end on days 17, 18 and 19, the infants were fed fruit or vegetable purée in our laboratory. On days 3–16, the parents fed their infants the fruit or vegetable purées at home.<br>Follow up at 12 and 23 months old using 3-day food diary reported by the parents	4-6 months	Mean vegetable and mean fruit intake increased significantly after the exposures. The mean intake of green beans and plums increased significantly after repeated exposure. The intake of the target food artichoke stayed low and the intake of apple only increased slightly.<br>Follow up: Reported daily intake of vegetables at 12 months of age was 38 % higher (P = 0.02) in the vegetable group (75 ± 43 g) than in the fruit group (54 ± 29 g) but was similar for both groups at 23 months of age (49 ± 43, 57 ± 35 g, respectively; NS).<br>Promoting vegetable intake during weaning, has a positive influence on vegetable intake until at least 6 months after weaning.	(+)	(Barends *et al.*, 2013, 2014)
1/day	9	Exposure vegetables: Carrot or a variety pack of courgette, parsnip and sweet potato<br>Novel vegetable: pea purée	Infants in the single taste group were given carrot puree (Ca) every day for 9 consecutive days, and infants in the variety group were given parsnip (Pa), courgette (Co) and sweet potato (Sp) with daily changes for 9 consecutive days.<br>All purees were made from fresh, organic vegetables, they were steamed, pureed, and boiled water was added as necessary to ensure a similar thickness that would be acceptable for young infants. No other	4-6 months	There was no main effect of exposure group (variety versus single taste) on consumption of a new vegetable.<br>There was an interaction between the age of introduction and exposure group on consumption of the new vegetable (pea). In particular, infants weaned at 6 months in the single taste group ate significantly less pea puree than those in the variety group.<br>This study showed that the efficacy of exposure may depend upon the age of the infant.	(+)	(Coulthard, Harris and Fogel, 2014)

			tastes or seasonings were added. The exposure pack vegetables were cooked, pureed, weighed, and placed into pots and then frozen.<br>On each day, the infants were fed at home; however, on days 1 and 11 the dyads were visited at home by the researcher. Mothers prepared the food in the normal utensils used to feed the baby, and the infants were fed in their normal feeding position.				
1/day for 12 days then 2/day for 12 days 1/d for 11 days	12 and 24 and 11	Vegetable purée: green beans, carrot, spinach, broccoli or parsnip served puréed, with added milk or added baby rice.	Vegetables were rotated daily. Spoons, small containers for measuring quantities of milk/rice/puree and a set of small digital scales was given to ensure the feeds were accurately prepared and recorded.	6-12 months	Intake, liking and pace of eating were greater for intervention group than control infants. However, at 6m then 18m follow up, vegetable (carrot > green beans) but not group differences were observed.<br>Infants previously exposed to vegetables with food ate on average 70% more of the plain vegetable purees and at a faster rate than those given no vegetables, although carrots were eaten and liked the most.	(+)	(Hetherington *et al.*, 2015)

Table S2: Studies using mere exposure to increase intake/liking in toddlers and children (>12 months -17 years). The studies are sorted according in ascending to publication year. Success-column: an evaluation of the effect of mere exposure; +: clear effect, (+): some effect but not clear, -: no effect.

Exposure Frequency	Number of Exposures	Exposure Stimuli	Serving Style	Age of Partici-pants	Results	Success	Author
**TODDLERS AND CHILDREN > 12 MONTHS -17 YEARS (EXCEPT 1 STUDY, WHERE 9MONTHS OLD ALSO PARTICIPATED)**							
1/day	20, 15, 10, 5, (2) or 0 (control)	Five initially novel kinds of cheese: Danish Esrom, Phila-delphia cream cheese, Norwe-gian gjetost, Nauvoo blue, and Wisconsin cheddar. Five initially novel fruits: ly-chees, dried apricots, kadota figs, Queen Anne cherries and dried bananas.	Small, bite-sized pieces, ap-proximately 0.75 cm on a side. Served at ambient tempera-ture.	2 years	Preference is increasing as a func-tion of exposure (positive and sig-nificant correlations)	+	(Birch and Marlin, 1982)
2/week	15	Tofu (plain, sweet or salted)		4-5 years	Preference increased for the ex-posed version only. Experience with 1 flavoured version did not produce generalised liking for all 3 versions of the food. Experience with 1 version (flavoured or plain) actually produced a decline in preference for the other version.	+	(Sullivan and Birch, 1990)
1/day	8	Red pepper	Small pieces of raw red pep-per (average weight 1.8 g)	5-7 years	Significant increase in consump-tion and liking of the stimuli com-pared with the control group	+	(J Wardle *et al.*, 2003)
1/day	14	Carrot, cucumber, red pepper, green pepper, tomato or celery.	Parents were asked to offer their child a taste of their target vegetable every day for 14 consecutive days. Suggestions to encourage tasting were offered (but no reward).	2-6 years	Intake and liking increased signifi-cantly for the target vegetable, but the intake was low (increased from 4,1-9,0 g)	+	(Jane Wardle *et al.*, 2003)

1/day	8	Sweet orangeade, sour orangeade and yoghurt which varied in balance of sweet and sour tastes.	Children: 200ml orangeade served in a cup at school	8-10 years	Children: Increased liking of sweet orangeade and sweet yoghurt, but no changes in liking of sour orangeade.	+	(Liem and de Graaf, 2004)
3/day	6	Carrot, cauliflower, broccoli, peas, zucchini or pumpkin. Vegetables ranked three and four (from one (most liked) to six (least liked)) were selected as the conditioned stimuli (CS) - paired with dextrose (CS+) or unsweetened (CS-).	Fresh vegetables were cooked separately until soft. The soft vegetables were mashed using a kitchen blender. Approximately 50 g of this mash was diluted in 100 ml water and poured into a 250 ml paper cup. The cup was sealed with an opaque plastic lid and a straw was inserted through the lid. Children were tested in small groups during school hours. Presented with two vegetable tastes (one paired with dextrose) at a time and instructed to sip and swallow from both drinks approximately every 5 min	4-6 years	Results show an increase in preference for the previously sweetened vegetable taste but not an increase in liking for the taste not being paired with dextrose.	**-**	(Havermans and Jansen, 2007)
1/day	14	The vegetable drinks: cucumber, carrot, iceberg lettuce, red (bell) pepper, plum tomatoes and beetroot. The drinks ranked three and four were chosen as target drinks	Juices were prepared with a juicer. Testing was done at school, morning break (10:15 h). When the children entered their classroom after playing outside, each child's table was equipped with one cup of vegetable juice (150	7-8 years	No mere exposure effect (or other effects) was seen, possibly due to too low vegetable juice intake.	-	(Zeinstra *et al.*, 2009)

			g), one cup of water for palate cleansing (150 g) and one piece of gingerbread (72 kcal). The children were instructed to drink as much of the vegetable drink as they wanted, preferably the whole cup.				
4/week	12	Variety of vegetables: carrots, peas, cauliflower, broccoli, red cabbage, beets (beetroot), French beans and spinach. Numbers 3 and 4 of the preference rank-order were selected as target vegetables.	Scheduled dinners at 5, 5:30 or 6pm with parents at a restaurant like setting. Meal of the day was presented and then brought by the research assistant. Tables in the restaurant were attractively set, with placemats, cutlery, and napkins, and background music was playing softly.	4-6 years	Found no difference in vegetable intake or liking.	-	(Zeinstra *et al.*, 2010)
1/week	10	Green bell pepper, carrot, peas, tomato	Food was served in the cafeteria with the regular school lunch. The hot and cold vegetables were served in separate containers One baby carrot, a small piece of tomato (1/32 of a medium sized tomato) and one-half tablespoon of diced green bell pepper, one-half tablespoon serving of cooked canned green peas without any seasoning.	4th– 5th grade students	Repeated exposure to initially disliked vegetables increases liking of 3 out of 4 vegetables (of those willing to taste the vegetables). The number of children who reported liking or liking a lot for previously disliked vegetables was greater after eight or nine taste exposures.	+	(Lakkakula *et al.*, 2010)

1/day	12	Disliked target vegetable: either carrot, red pepper, sugar snap pea, cabbage, cucumber, or celery	Small piece (2.5 g)	4-6 years	Increase in vegetable intake and liking was seen from pre- to post-intervention. However, the effects of exposure with no reward became nonsignificant by 3 months.	(+)	(Cooke *et al.*, 2011)
1/week	16 (4 months)	Carrots, celery and broccoli	Children received vegetables during a nutrition class presented with three varieties of raw vegetables that were premeasured in 1-oz portions and packaged in plastic bags.	11-13 years	Multiple exposures to vegetables alone resulted in reduced intake of vegetables in both children who reported eating vegetables and children identified as vegetable resistant.	-	(Johnston *et al.*, 2011)
2-3/week	9	Initially liked kamut snack bar, initially disliked Sea buckthorn snack bar	Bars were served in small white paper bags containing 5 bars of 40 (±2) g. Testing took part in the classrooms.	9-11 years	Significant increase in intake in both groups. Liking increased significantly of the disliked sea buckthorn bar to the same level as an already liked bar. No increase in the already liked kamut bar	+	(Hausner *et al.*, 2012)
2-3/week	10	Artichoke purée Control carrot purée	The purées were served in pre-weighted plastic cups at room temperature. Serving size: 100 g (Maximum two servings were offered per session corresponding to 200 g artichoke)	2-3 years	Significant increase in intake. Intake levels reached maximum at the 5th exposure that was sustained to the re-testing 6 months after the 10th exposure.	+	(Hausner, Olsen and Møller, 2012)
1-2/week	10	Cauliflower, snow peas, green pepper	Bite-sized pieces in a snack-size plastic bag with student identification. Served by the teacher at lunch time.	3-6 years	No effect of repeated exposure, but friends around the table were a significant predictor of vegetable consumption	-	(O'Connell *et al.*, 2012)
1/day	12	Cauliflower, sugar snap peas, celery and Chinese radish	Serving size: 100g Served in round, transparent plastic beakers (300 ml)	9-11 years	Exposure did not increase liking. Intake was stable. It decreased for some vegetables, but was affected by liking level of the vegetables	-	(Olsen *et al.*, 2012)

			with name and class of each child written on the lid. Sugar snap peas served whole, cauliflower in bushes, Chinese radish and celery in quadrangular sticks.				
1/day	8	Different fruit and vegetables each day	Apples, oranges, and bananas: whole<br><br>Other foods: bite-size pieces in pre-weighed, clear quart-size plastic, sealed freezer bag.	4-8 years	Increase in consumption of fruit but not vegetables	(+)	(Osborne and Forestell, 2012)
2/week	8	Steamed red pepper strips and steamed yellow squash coin	Very small portion (4 g) of steamed yellow squash coins or red bell pepper The tastings occurred in the morning, approximately one hour prior to lunch being served or in in the afternoon, immediately following naptime and before afternoon snack.	3-6 years	There was a significant increase in liking of the vegetable (served alone) from pre- to post-test.	+	(Anzman-Frasca *et al.*, 2012)
2/week	14 (2x7)	Soups of endive and spinach (half of each of them with high energy content)	Soups were consumed just before lunchtime as an entrée, followed by the regular lunch.	2-4 years	Exposure to the two vegetable soups (7 times each) did increase ad libitum intake of both independently of the energy content. This indicates a robust effect of mere exposure on intake. The intake of both soups was stable in 6-month follow up.	+	(de Wild, de Graaf and Jager, 2013)

1/day	2 weeks	Target vegetables included twenty-two different vegetables, the most common being carrot (n 20), cauliflower (n 17), cucumber (n 17) and broccoli (n 16).	Parents prepared (raw or cooked) vegetables providing half a cup of each in small pieces. Only addition of oil or butter was permitted.	4-6 years	Increased liking and intake of target vegetable was seen at post-intervention.<br>Target vegetable consumption continued to increase significantly over the follow-up period for Exposure + reward and control group but not for Exposure Only.	(+)	(Corsini *et al.*, 2013)
1/day	10	Artichoke purée (novel)<br>Carrot purée (control)	Offered mid-morning, prior to lunch (around 11.00 hours), or as a mid-afternoon snack (14.00 hours). Participants were offered pots of vegetable by the experimenters and nursery staff, who were familiar to the children. Children could consume as much or as little as they liked.	9-38 months	Intake of both vegetables increased over time; artichoke intake increased significantly more than carrot intake.<br>Five exposures were sufficient to increase intake compared to the first exposure.	+	(Caton *et al.*, 2013)
3/week	Program of 5 months	Four vegetables (acorn squash, baby spinach, cucumber slices, and sugar snap peas) and four fruits (pears, pink grapefruit, papaya, and kiwi).	Food was prepared for consumption (e.g., chopped or sliced) in 4 oz plastic containers.<br>All foods were presented raw, except for acorn squash (which was cooked).	5-6 years	More willing to taste fruits after the exposure, but not vegetables	(+)	(Schindler, Corbett and Forestell, 2013)
Not specified	Between 5 and 10	Artichoke purée (novel) in three versions (basic, sweet or added energy)	Children were given up to 200 g (2×100 g pots) of basic artichoke purée during a state of hunger, either before a main meal or as an afternoon snack or at the beginning of a meal.	4-38 months	Overall, younger children consumed more artichoke than older children. Change in target vegetable intake (post - pre intervention) was predicted by age and enjoyment of food, with younger chil-	(+)	(Caton *et al.*, 2014)

			The recipe, used for the repeated exposure condition and for the pre- and post-intervention measurement, was a basic artichoke purée (48 kcal/100 g). For the flavour-flavour condition the chosen unconditioned stimulus was sweetness (sucrose, 51 kcal/100 g) and for the flavour-nutrient condition, the chosen unconditioned stimulus was a higher energy density (addition of sunflower oil, 144 kcal/100 g)		dren and those with higher enjoyment of food scores being more responsive to repeated exposure		
5/week (3 ½ weeks)	7 x cauliflower 7 x Brussels sprout	Cauliflower and brussels sprouts	Brussels sprouts, halved, and cauliflower in florets added to boiling water and cooked for 5 and 7 minutes, respectively. Each child was served 15 g of each vegetable in blue, 3-oz cake moulds and a glass of water.	3 - 5 years	Exposure was increased liking for and intake of a cauliflower, but not for Brussels sprouts.	(+)	(Capaldi-Phillips and Wadhera, 2014)
2-3/week	6-8	Root vegetable purée: celeriac, swede and turnip	Purées were produced using organic frozen celeriac, swede and turnip with no other ingredients added and frozen as individual 100 g portions. Purées were offered to the children by experimenters or nursery staff, who had	15-56 months	Both exposure and flavour-flavour learning increased vegetable intake by 200 to 400% from pre- to post-intervention	+	(Ahern *et al.*, 2014)

			been instructed to approach feeding as they normally would. Children received up to 200 g of each vegetable purée at their usual snack time and they were asked to consume as much or as little as they would like.				
2/week	8	Salsify purée (target vegetable) Carrot purée (control)	At nursery, lunch time foods were presented warm as a starter at the beginning of the toddlers' lunch The sample jars were defrosted and heated (>63 °C) served at app 45°C Two 100 g jars of purée were available per child.	2-3 years	Intake of the target vegetable increased from pre- to post- exposure. Increase was significantly higher in the repeated exposure group. The increase of the target vegetable intake was still observed after 6 months.	+	(Bouhlal *et al.*, 2014)
1/day	4 x 4	1) apple and fennel; 2) pear and radish; 3) grapes and broccoli; 4) miyagawa and carrot.	Approximately 40 g of each fruit-vegetable combination was served raw, cut into standardised pieces of uniform size and presented at room temperature in plastic cups coded with the word "fruit" or "vegetable" Served at 10:30 am, immediately prior to the mid-morning break.	6-9 years	The intervention is effective in reducing food neophobia and this effect is also observed over the long term (6 months). Additionally, the program was successful in increasing liking for fruits and vegetables, although the effect was more pronounced for fruit. Systematic decrease of liking over time in the control group.	(+)	(Laureati, Bergamaschi and Pagliarini, 2014)
1/day	14	Baby corn, celery, red pepper, cherry tomato, cucumber or sugar snap peas	Raw vegetables chopped into approximately 2.5 g pieces (small enough to fit in the mouth) and served in 30 g portions	2-4 year	No significant differences in post-intervention liking or consumption of the target vegetable were found between the repeated exposure group and the control group.	-	(Holley, Haycraft and Farrow, 2015)

			Vegetables were provided by the research team. Parents were asked to conduct all offerings outside of a mealtime and instructed to simply offer their child a small piece of the target vegetable without eating it themselves and to remain neutral in their responses to whether or not their child tasted the piece.		Repeatedly offering in a neutral way did not appear to ensure tastings in this study.		
2-3/week	8	Sea-buckthorn or aronia juice	Testing took place in classroom between 9-12am. Juices were served in cardboard cups with black lids and black straws. Serving size was approximately 50 g for aronia and 90 g for sea-buckthorn juice, if they finished one cup, they could ask for another.	9-11 years	Liking did not develop significantly for any of the juices across exposures while it developed similarly for both juices. A significant increase was found for the 'initial dislikers' only. Intake of sea-buckthorn juice increased significantly over the eight exposures and remained high after 6 months Intake of aronia juice was only increased at follow-up sessions.	(+)	(Hartvig *et al.*, 2015)
2/week	14	Red beet and parsnip crisps	Freeze-dried vegetable crisps with a dip. Raw, fresh vegetables were used to prepare vegetable crisps. Vegetables were blanched and further processed in a professional freeze-dryer. Products were sealed in bags of either 6 g each (used in conditioning	1.5-4 years	Intake increased significantly after the intervention for both vegetables (on average with 8 g; an increase of approximately 300%), and this effect was persistent even 6 months afterwards.	+	(de Wild, de Graaf and Jager, 2015)

			period) or bags of 30 g each (used in pre-tests and all post-tests).				
1/week	6	Plain spinach, pureed spinach and spinach ravioli. Control: green beans	A randomized controlled trial with four parallel groups: plain spinach, creamed spinach, spinach ravioli, and green beans. During the intervention, children were served the vegetable at their main meal six times over 6 weeks at home. The vegetable products were provided by the researchers.	2-4 years	All four groups significantly increased their spinach intake from pre to postintervention by an average of 70%. For preference, no significant shift toward the target vegetable was found from pre- to postintervention. The effect on intake depended on the child's neophobia status and preintervention spinach consumption, with children with neophobia being less responsive to the intervention and with children who ate more spinach before the intervention being more responsive to the intervention.	+	(de Wild, de Graaf and Jager, 2017)
-	1	3 desserts (in balanced order): smooth strawberry yoghurt, strawberry yoghurt with pieces and strawberry jelly	The desserts were presented in equal clear plastic bowls (8 cm). The children were asked to taste a spoonful of the food and if they tried it, the experimenter asked if they would like another spoon. The children handled the spoons themselves, the experimenters supervised if the spoons were properly filled and helped the younger children when needed.	3-10 years	Results showed that the children in the exposure condition ate specifically more of the jelly dessert - the texture of which they had been pre-exposed to - compared to the children in control condition. No group differences were found for the other two desserts.	+	(Nederkoorn *et al.*, 2018)

12/5 months	12	Pumpkin, courgette, and white radish	Pumpkin blanched and as a cracker spread; courgette blanched and as soup; white radish raw and as a cracker spread. ~20g of pumpkin and courgette ~10g of white radish	0-4 years	There was a significant positive effect of the intervention for pumpkin and white radish. Results for willingness to taste were in the same direction. There was no repeated exposure effect for courgette.	(+)	(Zeinstra, Vrijhof and Kremer, 2018)
1/week	12	Raw mooli	Taste exposure at snack time. The researcher prepared mooli as snacks, delivered this to each preschool, and the snacks were provided to children by preschool staff members. The vegetable snack was offered in pre-weighed 40-g portions using individual snack bags labelled for each child.	2-5 years	Repeated taste exposure significantly improved intake of the target vegetable. Taste Exposure increased intake from 4.7±1.4 g to 17.0±2.0 g and this was maintained at both follow-ups.	+	(Nekitsing *et al.*, 2019)
2/week	5-6	Three target vegetables (baby sweet corn, celery and red pepper). To ensure variety, a further 2 vegetables, radish and green pepper, were also selected to be included in the mixed vegetable snack based on the same criteria (familiar, but were not typically consumed as snacks)	All children were offered a bag containing slices of a single vegetable (their assigned target vegetable) and a bag containing a variety of sliced vegetables on two separate days and this was counterbalanced to avoid order effects. All snack sessions were carried out by nursery staff although a researcher was present for the first session at each nursery. The single	24-55 months	Vegetable intake increased significantly from pre to post intervention for snacks congruent to the condition to which children were assigned. Magnitude of change was smaller for the variety condition. Follow up data revealed that snack intake remained significantly higher than baseline 1-month post-intervention.	+	(Ahern *et al.*, 2019)

			vegetable snack consisted of 100 g of one of the three target vegetables (baby sweet corn, celery or red pepper). The variety snack was a mix of 20 g of each of the five vegetables (baby sweet corn, celery, red pepper, green pepper and radish).				
1/day	15	Fruits and vegetables (disliked or unfamiliar to the child)	Parents were asked to offer children a taste of both foods every day for 15 consecutive days. Families received home deliveries of the target foods three times during this period (on approximately the 1st, 6th and 11th day of exposure); each delivery provided enough for at least five servings. Fresh foods were provided where possible, but tinned, frozen or dried foods were substituted if fresh foods were not available. Parents were asked to provide a child-sized portion of each food every day, defined as the amount that would fit into the palm of their child's hand	18-24 months	The total number of exposures provided was not a robust predictor of children's acceptance of foods or of parents' positivity towards the intervention, likely due to parents' broad adherence to the instruction to offer both foods every day.<br>Children's willingness to taste and intake of the targeted vegetable, and their liking of both the target fruit and vegetable, increased with repeated opportunities to taste these foods. However, parents as a group did not find taste sessions to become easier or more enjoyable over the course of exposure.	+	(Houston-Price *et al.*, 2019)

1/day	10	Turnip (target vegetable)	Steamed-pureed turnip	3-5 years	There was a significant effect of exposure shown by significant increases in intake ($p < 0.001$) and liking ($p = 0.008$) post-intervention	+	(Mohd Nor *et al.*, 2021)

Table S3: Studies using mere exposure to increase intake/liking in young adults and adults (> 18 years). The studies are sorted according in ascending to publication date. Success-column: an evaluation of the effect of mere exposure; +: clear effect, (+): some effect but not clear, -: no effect.

YOUNG ADULTS AND ADULTS >18 YEARS							
**Exposure Frequency**	**Number of Exposures**	**Exposure Stimuli**	**Serving Style**	**Age of participants**	**Results**	**Success**	**Author**
All the samples were presented on the same day and the test was repeated one week after	20, 10, 5, 0	Four novel tropical fruit juices	35 25-ml cups, each containing 7 ml of juice	Under-graduates	Within study (all participants tasted three juices 20, 10 or 5 times), the higher the number of exposures, the more liked are the juice. Liking ratings increased monotonically with increasing exposure.	+	(Pliner, 1982)
1/week	4	Two experimental foods: Spinach Mousse and Beef and Vegetable Jelly, prepared either without MSG or with 0.3 to 1.2% MSG added	On test days, the experimenters added MSG to target foods whenever appropriate. This was done covertly, and the subjects were unaware of the manipulation. Foods were weighed before being presented to the subjects. The subjects ate freely, under habitual conditions. After the end of the meal, food left-over by each individual subject was weighed. Weekly tests of free intake were then carried out under laboratory conditions for four consecutive weeks.	young men and women	On the first presentation, foods with 1.2% MSG added induced large meals and fast eating rates, two indices of immediate palatability. Over repeated weekly exposure subjects fed the test foods with 0.6% added MSG progressively ate more and faster, revealing an increasing stimulation to eat with repeated exposure. In the group exposed to the experimental foods without MSG added, free intake either remained stable or declined over the four weekly tests.	(+)	(Bellisle *et al.*, 1991)
1 / second week	2 x 6	Two lunch menus were selected. The menus were of medium palatability and included a	All foods included in the menus were familiar to the subjects. For the duration of the experiment, they were not served except on test days. The subjects ate freely, under habitual conditions.	Elderly	Effect of MSG on energy intake during the test meals appeared only for Menu B. Higher intakes of soup and mashed potatoes, the target foods to which 0.6% MSG was added.	+	(Bellisle *et al.*, 1991)

		soup and a vegetable dish with added MSG					
1/week	5	Ratatouille containing capsaicin at either 2.5 or 5"0 ppm	Subjects were presented with two portions of 195 g of canned ratatouille preheated in a microwave (serving temperature approx. 65°C) as an experimental meal during lunch hour. The second portion of ratatouille contained either 2.5 or 5.0 ppm capsaicin	Students and employees of the University of Sussex	This experiment provides the first evidence that repeated exposure to capsaicin enhances liking for the chilli burn	+	(Stevenson and Yeomans, 1995)
1/day	8	Sweet orangeade, sour orangeade	Adults: 200ml to drink from a specific cup to drink at home	20-24 years	Adults: No changes neither for sweet nor sour orangeade	-	(Liem and de Graaf, 2004)
1/week	5	Either peas, spinach type 1, spinach type 2 or a cream spinach recipe, containing cheese and spices	Products were presented to the consumers unbranded in plastic bags along with cooking, serving and eating instructions. The products were preweighed at Unilever to achieve portions of 300g. The respondents had to eat at least one quarter of the food themselves.	18-64 years	Repeated consumption of plain spinach did result in an increased liking for plain spinach overall, specifically for spinach dislikers. Consumers who initially disliked spinach showed an increased liking for spinach after having repeatedly eaten it in the learning phase as part of a recipe with cream, cheese and spices.	+	(Bingham, Hurling and Stocks, 2005)
19/6 weeks	19	Earl Grey decaffeinated black tea sweetened with sucrose at various concentration l evels (Low or optimum)	Test/exposure in a room decorated as a tearoom morning (8-12) or afternoon (1:30pm-4pm) The tea was served at approximately 70 °C. Tea was served in Styrofoam cup containing 350 ml of tea with assigned sucrose concentration. Respondents were asked to drink as much tea as	University staff and students	Liking of the low sweet tea increased significantly from session 1 to sessions 10–19, whereas the liking of the optimum sweet tea showed no trend towards changing over the sessions.	+	(Chung and Vickers, 2007)

			they wanted; they could request more.				
1/week	24 over six exposure sessions	Uncommon fruit drinks: melon and anise, apple and orange blossom, apple and verbena, tamarind juice	Testing was done in a laboratory setting. Each session, the participants received a series of 16 samples – 4 samples of each drink, 20 ml per sample – coded by a three-digit number.	20–78 years	Results showed that exposure led to an increase of liking for drinks with a high arousal potential, while no evolution of liking was observed for drinks with a moderate arousal potential but a higher sweetness intensity.	(+)	(Sulmont-Rossé *et al.*, 2008)
1/day or every 3 days	10 sessions	Green tea drink optimal sweetness level (OP) and low sweetness level (LO)	50ml of each sample was served in a transparent glass container coded with a 3-digit random number. The subjects were instructed to consume the entire amount of the drink they received. (1-day intervals or 3-day intervals)	18-49 years	Repeated consumption led to an overall increase in liking of the novel drink.	+	(Son, Hong and Kim, 2010)
1/day	2 x 6	Sports drinks with six different acidulants added at equal sour concentrations.	The drink was given in translucent unbranded polypropylene 500 ml sports drink bottles to consume at home.	University staff and students	Products that were initially preferred became less preferred after 14 days.	-	(Kinnear and de Kock, 2011)
Approximately 1/day	8	Carrot and coriander soup produced in the laboratory with and without salt.	Exposure took place prior to lunch Three groups: 20 ml soup samples with no added salt, 280 ml bowl of no-salt soup, or to a control group that received 20 ml soup samples containing salt at 280 mg/100 g (within normal, commercial range).	20-55 years	The two groups receiving the no added salt soup showed increases in liking starting at the third exposure, this was also evident in a repeat assessment following the exposures	+	(Methven, Langreney and Prescott, 2012)

2/week	20	Quorn product (meat like)<br>Tofu (not meat like)<br>Meat reference (chicken fillet)<br><br>All products commercially available	Participants received cooled test product to prepare at home and were instructed to use the product as a meal component within a hot meal. Preparation guidelines: stir frying for 5 min in sunflower cooking oil in a separate cooking pan.<br>The minimum amount to consume was one third of the provided amount (50 g). ´	18 - 66 years	On a product group level, boredom occurred with all three products and after 20 exposures there were no significant differences in product liking anymore. However, there were noticeably different individual responses within the three product groups, showing both 'boredom' and 'mere exposure' patterns. Mere exposure occurred significantly more frequent with tofu, with more than half of the participants showing an increased liking over time.	+	(Hoek *et al.*, 2013)
1/day	84	Spicy, honey roasted, roasted and unsalted, or roasted and salted peanuts.	Participants incorporated 42 grams of peanuts into their diets each day for 12 weeks.	18-50 years	Peanuts were generally well-liked in the study, with a mean liking of 69 on a 100-mm visual analogue scale and a decrease of less than 15% over 12weeks.	-	(Jones *et al.*, 2014) (Not a mere exposure study)
1/day	3	Tomato soup: plain, low salt or low salt with added herbs and spices.	Soup samples were prepared and held in a water bath at 70 °C before serving at 65 ± 3 °C. Respondent received 30mL soup in a china cup (50 mL). For the repeated exposure (Visits 3–5), tomato soups were prepared and then held in thermos flasks for a maximum 15 min before serving, at 69 ± 4 °C; each participant received a full portion (400 mL) of soup.<br>Laboratory setup	35-60 years	Repeated exposure to the herbs and spice-modified soup led to a significant increase in the overall liking and liking of flavour, texture and aftertaste of the soup, whereas no changes in liking were observed for the standard and low salt tomato soups over repeated exposure. Moreover, a positive trend in increasing the post-exposure liking of the herbs and spices soup was observed, although this was not significant.	(+)	(Ghawi, Rowland and Methven, 2014)
16/week	4 x 16	Biscuits, a standard variant and 4 variants differing by the level of reduction of either	In laboratory: Tasting was same time each week either morning (7:30-9:30am) or afternoon (5-7pm) Participants were asked	19-62 years	Exposure to the most fat-reduced variant increased the liking for this specific variant to a level equivalent to the standard variant.	(+)	(Biguzzi, Lange and Schlich, 2015)

		fat (33%) or sugar content (28%).	to consume each biscuit entirely. At home: Biscuits were packaged in four transparent plastic bags of four biscuits (~12.5 g each). Participants could consume biscuits when and where they wanted.		Exposure to the sugar-most-reduced variant did not increase the liking for that variant but did increase liking for the first two intermediate reduced-variants. No evolution of liking was observed exposure to a standard variant. However, the sugar-most-reduced variant was significantly less liked after exposure to the standard variant		
8/4 weeks	8	Natural cheese: Brie, Emmental, Gorgonzola, Gouda, Parmigiano-Reggiano and sharp Cheddar.	The cheese samples were kept at 4 °C in a refrigerator, and then allowed to equilibrate at room temperature for 1 h prior to each experiment. All of the cheeses were cut into small cubes (2.8–3.0 g) that were placed in disposable cups (diameter of 7.0 cm and height of 4 cm; Samboopack, Gyeonggi-do, Korea) which were labelled with 3-digit random numbers for the evaluations. All cheeses were served in raw state.	Female subjects (age not specified)	In the repeated exposure tests, Brie, Gouda and sharp Cheddar cheese samples were preferred over Emmental, Gorgonzola and Parmigiano-Reggiano cheese samples throughout the eight exposure sessions. The liking of Brie, Emmental, Gouda, sharp Cheddar cheese tended to increase over time, whereas it remained constant for Gorgonzola and Parmigiano-Reggiano This study has shown that the acceptance of natural cheese flavors can develop as consumers are repeatedly exposed to them, but the level of acceptance may vary with the type of cheese.	(+)	(Go, Kim and Chung, 2017)
Not specified	4	Kangkung (Indonesian-style stir-fried spinach) seasoned with four flavour variants of Korean fermented soybean paste: sweet, umami, hot and spicy, and fermented.	The food samples were cooked one hour prior to each consumer taste test and exposure sessions. 16.5–17.5 g of each sample (55–65 °C) was served in a lidded disposable plastic cup coded with a three-digit random number. Subjects were asked to freely taste as much as they wanted for the taste-test-trials but were encouraged to	18-58 years	The hedonic and perceptual discrimination abilities of the samples increased for the entire group as the repeated-exposure sessions progressed. Moreover, overall liking score increased with exposures for most of the samples. It was evident that repeated exposure influenced the acceptability and discrimination ability of kangkung samples among the consumers.	+	(Song *et al.*, 2019)

			taste all the samples served for the repeated exposure sessions.				
4/3 days	4	Wine products	The sensory tests were conducted in a dedicated tasting room (ISO 8589: 2010). Wines (25 mL) were served at room temperature (19 °C), in ISO glasses (ISO 3591: 1977), coded with three digits.<br>The consumers in group 2 (repeat-exposure group) received two bottles of wine, labelled A and C. They were requested to drink these wines, alone, before meals, four times over the three days, and to randomize the order of consumption (both wines were tasted on each occasion)	20-over 50	The experiments revealed that daily exposure modified consumer appreciation of wines with different fruity profiles<br>Considering the results obtained for both groups, familiarization with the wines modified the consumers' hedonic perception and WP for repeat-exposure wines.	+	(Tempere *et al.*, 2019)
1/day	15	Flavoured beverages<br>horchata, lulo, yuzu, papaya, chamomile, aloe vera, mamey, and maqui berry	340 mL of the beverage that contained a non-nutritive sweetener, sucralose (0.0078% w/v), citric acid (0.1% w/v) food colouring (red; McQueen, Sainsbury's UK) and filtered water.<br>To standardise at-home consumption as much as possible, participants were asked to: 1) store the beverages in a refrigerator to preserve beverage flavour and integrity; 2) to shake the chilled beverages for 15–20 s before consumption; 3) to	18-70 years	Over the exposure period, we observed an increase in liking for all test (CS+) flavours relative to untested (CS−) flavours.	+	(Attuquayefio *et al.*, 2020)

	drink the beverages at approximately the same time every day; and 4) to consume them within 10 min in isolation (i.e., without food) within a 20 min window prior to or after a meal.	

Table S4: **Studies using mere exposure to increase intake/liking (recalled/retrospective data).** The studies are sorted according in ascending to publication date. Success-column: an evaluation of the effect of mere exposure; +: clear effect, (+): some effect but not clear, -: no effect.

RECALLED/RETROSPECTIVE DATA							
**Exposure Frequency**	**Number of Exposures**	**Exposure Stimuli**	**Serving Style**	**Age of Participants**	**Results**	**Success**	**Author**
Recalled/early life exposure to sweetened water and salt during the first two years of life. Frequency (sweet): never fed, fed for six months or less, fed for more than six months Frequency (salt): salt in cooking, no salt in cooking			Stimuli used for testing preference: Sucrose solution Sweetened or plain Kool-Aid Salted and unsalted carrots and carrot soup and salt crystals.	2-year-olds that have been previously tested at 6 months	Children who had been regularly fed sugar water by their mothers consumed more sucrose solutions but not more water than did children whose mothers did not feed them sugar water. Prior exposure to sugar water was unrelated to consumption of sweetened or unsweetened fruit-flavoured drink. Salt dietary exposure was unrelated to individual differences in acceptability of salty foods.	(+)	(Beauchamp and Moran, 1984)
Recalled exposure	Frequently, rarely, never	Range of different foods: fruits, vegetables, meat, dairy and sweets/snack.		University students, age 18 and up	Subjects who recalled eating vegetables even occasionally in childhood showed a greater current liking for those foods, as compared to those who never ate them.	+	(Wadhera *et al.*, 2015)

Table S5: **Studies using visual mere exposure to increase intake/liking).** The studies are sorted according in ascending to publication date. Success-column: an evaluation of the effect of mere exposure; +: clear effect, (+): some effect but not clear, -: no effect.

VISUAL EXPOSURE							
**Exposure Frequency**	**Number of Exposures**	**Exposure Stimuli**	**Serving style**	**Age of Participants**	**Results**	**Success**	**Author**
1/day for 5 minutes Experiment 2: 1/day experiment 3: 1/day 5 mins	7, 14 or 21 Experiment 2: 14 Experiment3: 14	Experiment 1: Picture books all About Asparagus or All About Aubergine. Only pictures of asparagus or eggplant.	Parents were asked to read with their child for 5 minutes each day. (some parents were also told to talk	~2 years	Results show that positive preferences for stimuli are easily and reliably induced in children and, importantly, that this effect of exposure is not restricted to the exposed stimulus per se but also applies to new representations of the exposed item	+	(Houston-Price *et al.*, 2009)

		Experiment 2: enriched exposure book with pictures of 6 different fruits showing a variety of pictures of the fruits) and perceptual exposure book with 6 different fruits (same picture of the fruits shown in different sizes). Experiment 3: "a fruit book" with pictures of 8 unfamiliar fruits or "a vegetable book" with pictures 8 unfamiliar vegetables	with their child about the vegetable).		Children's visual preferences can be enhanced through repeated visual exposure to stimuli by parents in a naturalistic picture book setting Our data demonstrate that repeated exposure to stimuli in picture books leads young children to preferentially attend to exposed items over nonexposed items. Importantly, this effect is not limited to the perceptual stimuli seen during the exposure phase; rather, it generalizes to new depictions of exposed items		
~1/week	8	Coloured beetroots, cauliflowers, carrots, bell peppers and tomatoes	a 2-week visual exposure phase where place mats with pictures of vegetables were set on tables in school cafeterias. Three experimental conditions: simple exposure condition, diverse	3-6 years	Results indicated that visual exposure led to an increased consumption of exposed and non-exposed vegetables after the intervention period. Nevertheless, the exposure intervention where vegetables varying in colour were shown to children was no more effective.	(+)	(Rioux, Lafraire and Picard, 2018)

			exposure condition and control condition.				
1/day	15	Fruits and vegetables (disliked or unfamiliar to the child)	Each book contained 8 pages of colour photographs and information about the food in the format of a simple 'farm to fork' photo story showing how the food grows, how it appears in shops, and what it looks like when it is cut open, prepared and served. Parents were asked to look at the book with their child for a few minutes every day for two weeks.	18-24 months	Prior visual familiarization enhanced children's willingness to taste and liking of their target vegetable during repeated taste exposure and made the process of introducing vegetables easier and more enjoyable for parents. There were no effects of prior visual familiarization to fruits.<br>Children's acceptance of foods and parents' positivity during exposure predicted children's liking and intake of foods 3 months later. Results confirm the potential for vegetable picture books to support parents in engaging with repeated exposure regimes and in successfully introducing vegetables into toddlers' diets.	+	(Houston-Price *et al.*, 2019)
-	1	Mikado chocolate covered biscuit sticks	During session one, in the 'smaller portion size' condition participants	Mean: 23.6 years	Participants that were exposed to a smaller, as opposed to larger portion size subsequently believed that a normal portion of the snack food was smaller in size. Exposure to the smaller as opposed to the larger portion size also resulted	+	(Robinson *et al.*, 2019)

	were provided with a serving of 6 biscuit sticks (14g) and in the ‘larger portion size’ condition participants were shown a serving of 16 biscuit sticks (37g), both on the same standard white side plate.	in participants consuming less snack food the next day.	

Table S6: **Studies using hands-on mere exposure to increase intake/liking.** The studies are sorted according in ascending to publication date. Success-column: an evaluation of the effect of mere exposure; +: clear effect, (+): some effect but not clear, -: no effect.

HANDS-ON EXPOSURE							
**Exposure Frequency**	**Number of Exposures**	**Exposure Stimuli**	**Serving style**	**Age of Participants**	**Results**	**Success**	**Author**
2/week	9 (children were exposed to each one of the four foods five times across the nine different exposure times)	Two fruits (cantaloupe and nectarines) and two vegetables (green bell peppers and tomatoes), relatively less liked	At each exposure session, children worked together to follow the day's recipe and assemble the snack, using child-safe knives to cut the fruits and vegetables. Once complete, each child was given a serving of the prepared snack, and children sat together at classroom tables to eat.	6-8 years	Preferences for target foods (tomatoes, bell peppers, cantaloupe, and nectarines) increased from pre-test (Median = 5.8) to post-test (Median = 5.5; $p < 0.05$). When examining changes in preferences for target vegetables and target fruits separately, results were not statistically significant ($p = 0.11$ and $p = 1.0$ respectively).	(+)	(Ehrenberg *et al.*, 2019)
